# Abstracts from Foreign Journals

**Published:** 1900-08

**Authors:** 


### ABSTRACTS FROM FOREIGN JOURNALS.

Mumps in the Dog. By Busquet and Boudeaud. Translated from the *Comptes Rendus de la Société de Biologie*, vol. lii., No. 25, July 13, 1900, by Mazÿck P. Ravenel, M.D. (From the Laboratory of the State Live Sock Sanitary Board of Pennsylvania.)

Mumps, by which is meant a specific swelling of the salivary glands, and especially the parotids, is a very rare disease in the dog. Medical literature seems to be limited to cases observed by Schüssele (1842), Whitaker, Hertwig, and Busquet.

Recently, we have been able to prove: 1st, that the dog is susceptible to mumps; 2d, that the disease can be transmitted from dog to dog; 3d, that one finds in the sick dog a coccus which in the saliva grows as a diplo-streptococcus, analogous or identical with that found in man in cases of mumps by Féré and Busquet in 1895, and in the blood as a diplococcus which is analogous or identical with that found in man by Laveran and Catrin in 1893.

On December 8, 1897, a gentleman noticed a marked change in the appearance of his dog. The animal was dull, and refused food. In the evening frequent chills occurred. Next day the dog remained lying down, ate very little, and showed signs of obstruction in the nares, sneezing frequently. On the 12th the right parotid was swollen and hard, reaching the size of a small hen's egg, the lobules being clearly defined. The right submaxillary lymphatic glands were enlarged, and the skin over the region œdematous. Pressure over the area caused the animal to cry. The mucous membrane of the mouth was dry and saliva scant.

A diagnosis of mumps was made. By the 14th there was improvement, the animal ate and was brighter, the swelling persisting, however. This day the buccal mucous surface was disinfected with great care, and saliva drawn direct from the duct of Steno, with which cultures were made on various media. In all cultures a diplo-streptococcus was found, in pure culture in some tubes and mixed with a diplococcus and a staphylococcus in others. Inoculation of dogs was not practicable, unfortunately, and rabbits and guinea-pigs gave no results.

The dog had been in a room where it had access to a young fox-terrier of ten months, with which it had played. On the 14th this puppy was found playing with the cotton-wads which had been used to wipe out the mouth of the one with mumps. Two days later it sneezed frequently and had a rough cough, but remained bright and ate heartily. On the morning of the 17th there was a swelling in the right parotid region as large as an orange, which began at the base of the ear and descended behind the posterior border of the right inferior maxilla and went forward almost to the angle of the mouth. The parotid gland was swollen, and the duct of Steno stood out like a cord and was very hard to the touch. There was not much evidence of pain except on pressure. The skin over the right parotid and submaxillary regions was slightly œdematous. The buccal mucous membrane was dry and somewhat discolored; saliva scant. The general condition was little disturbed; the appetite scarcely affected. By the 19th the swelling and œdema had abated sensibly, the enlargement of the gland diminishing progressively until the 24th.

Cultures were made on the 18th from blood drawn from a vein and from saliva taken from the duct of Steno. The same organisms were found as in the first dog: diplo-streptococci, diplococci, and staphylococci in the saliva, and diplococci in the blood.

In both animals there was the same onset and the same clinical evolution of the disease, after three or four days of incubation dulness, general fatigue, loss of appetite, frequent chills, snuffling, and much sneezing; soon the cough appeared, about the same time as the swelling of the salivary glands, especially the parotid and submaxillary. The lobes of the glands can be easily made out. The skin over the region of the affected glands is œdematous and somewhat painful. Steno's duct is swollen, hard, and stands out like a cord. The corresponding lymphatic glands become affected early, from the third to fourth day. The buccal mucous surfaces are dry and somewhat discolored, and the flow of the saliva is scant. Mastication of hard matter is slightly painful. The general condition, except for a manifest fatigue, does not seem to be much affected. The entire course of the disease is about twelve days.

The authors conclude that there is in dogs an infectious disease which is localized in the salivary glands, and which may be transmitted from one animal to another. The disease corresponds entirely with what is known as mumps in man. The bacteriology will be worked out more thoroughly by the authors and reported on later.

